# Developing the biomarker panels and drugs by proteomic analysis for autoimmune uveitis and posterior scleritis

**DOI:** 10.1016/j.isci.2024.111389

**Published:** 2024-11-14

**Authors:** Xueru Li, Jinying An, Lingzi Wu, Qingqin Tao, Hui Zhang, Xiaomin Zhang

**Affiliations:** 1 Tianjin Key Laboratory of Retinal Functions and Diseases, Tianjin Branch of National Clinical Research Center for Ocular Disease, Eye Institute and School of Optometry, Tianjin Medical University Eye Hospital, Tianjin, China; 2 Beijing Institute of Ophthalmology, Beijing Tongren Eye Center, Beijing Tongren Hospital, Capital Medical University, Beijing 100730, China

**Keywords:** Health sciences, Medicine, Medical specialty, Immunology, Pharmacology, Natural sciences, Biological sciences

## Abstract

Autoimmune uveitis and posterior scleritis are ocular diseases caused by immune dysregulation. Their pathogenesis remains elusive, and delayed diagnosis can exacerbate vision loss. Our study analyzed proteomic profiles of 190 patients with Behcet’s disease uveitis, posterior scleritis, and Vogt-Koyanagi-Harada syndrome. Bioinformatics methods revealed potential pathogenesis and biomarkers for the diseases, which were verified by enzyme-linked immunosorbent assay. The diagnostic accuracy was improved by constructing a biomarker combination. In addition, we used the Connectivity Map tool to analyze the differentially expressed proteins and identified small molecules with potential clinical applications. In this study, EMINIL1 and LYZ were identified as biomarkers for Behcet’s uveitis, GSTP1 and PGLYRP1 for posterior scleritis, and APOH and STXBP1 for Vogt-Koyanagi-Harada syndrome. This study mapped the plasma proteins of these diseases, revealing potential pathogenesis and clinical applications of these biomarkers.

## Highlights

• Three disease plasma protein maps were constructed
• XGBoost model distinguished groups and assisted in identifying candidate biomarkers
• Identified two biomarkers per disease and formulated targeted biomarker combinations
• Screened out small-molecule drugs with potential clinical applications

## Graphical abstract

**Figure:**
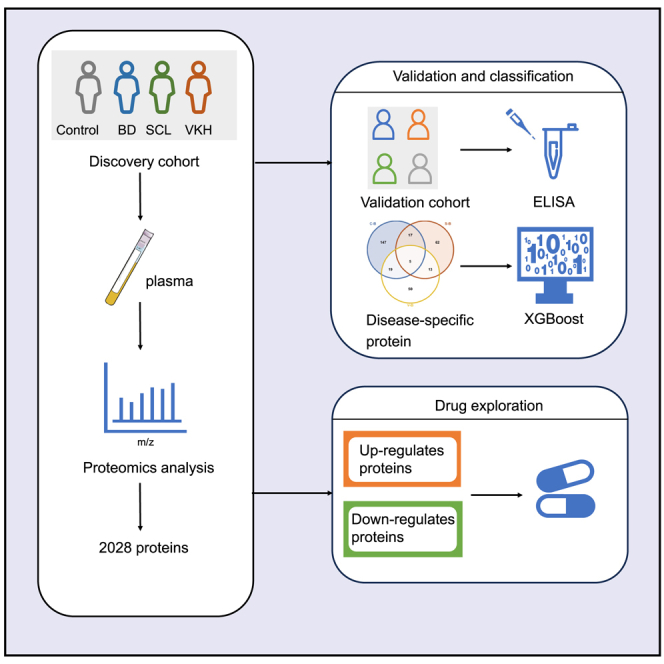

## Introduction

Autoimmune uveitis and posterior scleritis are intraocular inflammatory diseases characterized by recurrent inflammation of the retina and uvea.^1^ If not promptly diagnosed and treated, these conditions pose a threat to vision and can lead to vision loss. According to the Uveitis Clinic, approximately 20–70% of patients experience severe vision loss (best-corrected visual acuity ≤20/50).^2^ Typically, the onset of the disease is associated with systemic autoimmune disorders, such as Behcet’s disease (BD), Vogt-Koyanagi-Harada syndrome (VKH), and ankylosing spondylitis.

BD and VKH are the most common types of uveitis in China, with prevalence rates of approximately 8.7–12.7%.^3^ In BD, most patients present with bilateral ocular involvement, involving the anterior, posterior, and panuveitis. Panuveitis and retinal vasculitis are the most commonly observed forms.^4^^,^^5^^,^^6^^,^^7^ Although the exact cause of the disease remains unclear, studies suggest that immune system dysregulation plays a significant role in disease progression. This dysregulation leads to a large number of immune cells attacking tissues under the influence of inflammatory cytokines.^8^ VKH is a type of bilateral simultaneous or sequential granulomatous panuveitis, often accompanied by clinical manifestations involving the central nervous system, auditory system, and skin. The main pathological change in VKH is diffuse stromal choroiditis, with the inflammation that can extend to the choroidal capillaries, retina, and vitreous.^9^ Frequent episodes and persistent inflammation can affect the intraocular blood supply and lead to structural damage.^10^ In cases of scleritis, posterior scleritis (pSCL, abbreviated as SCL in subsequent chapters) accounts for approximately 2–12% of cases and is more common in females, although the reason for this disparity remains unclear (62.3%). SCL can occur at any age and typically affects only one eye. Clinical presentations of the disease vary widely and commonly include pain accompanied by decreased vision. It can also lead to retinal vein dilation, macular edema, and retinal vascular obstruction.^11^^,^^12^ Patients aged >50 years have a significantly higher risk of developing associated systemic diseases.^12^

In recent years, with advancements in uveitis research, increasing attention has been focused on the application of proteomic technology. Blood, is an easily accessible and non-invasive sample, making it an ideal choice for proteomics research. Previous studies have demonstrated that healthy individuals exhibit distinct and stable proteomic profiles in their plasma,^13^ further sparking interest in the potential of plasma proteomics for discovering biomarkers. The application of liquid chromatography-tandem mass spectrometry in proteomics enables comprehensive analysis of the expression patterns of the plasma proteome, providing insight into physiological and pathological states.^14^ The holistic view of proteomics allows the unbiased discovery of novel biomarkers without the need to predefine target proteins. This approach deepens our understanding of disease biology and facilitates the identification of new biomarkers, especially in cases where the characteristics of the diseases are not fully understood.

Currently, the diagnosis of the diseases mainly relies on clinical features and routine examinations. Treatment typically involves a combination of corticosteroids, immunosuppressants, and biologics to protect eye tissues and prevent excessive immunosuppression. However, as the disease progresses, each patient’s immune response and response to drug treatment can vary. We collected 190 plasma samples from healthy control (HC), BD, SCL, and VKH for proteomic analysis based on SWATH-MS. We identified potential biomarkers for each disease using bioinformatics combined with machine learning. We also explored the potential mechanisms of the disease by studying protein changes during its progression. This approach aims to promote the realization of precision medicine goals. Finally, we used different protein changes in the disease to screen available drugs, providing additional references and new ideas for disease treatment.

## Results

### Clinical features and participant distribution

**Figure 1:**
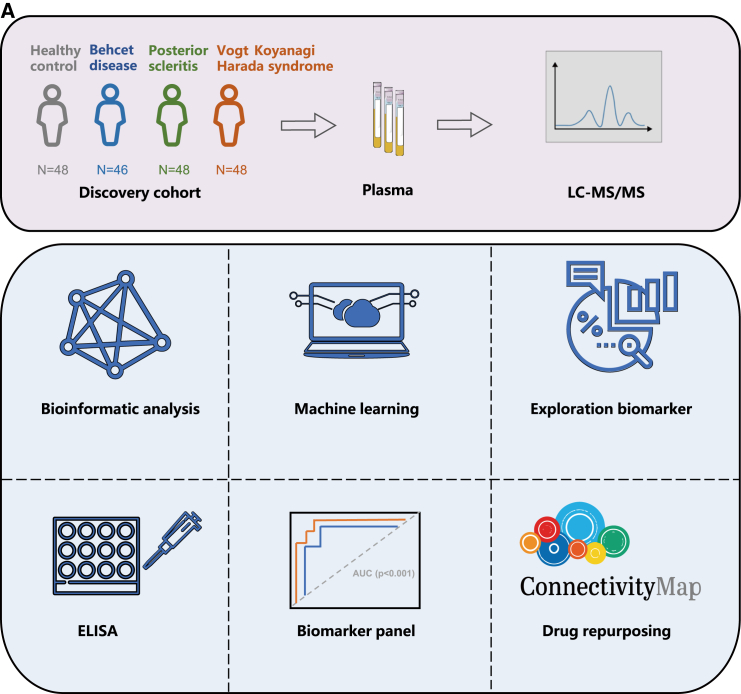
Workflow diagram and participants clinical information (A) A total of 190 subjects were included in the study, comprising healthy control (HC), Behcet’s disease (BD), posterior scleritis (SCL), and Vogt-Koyanagi-Harada syndrome (VKH). Proteomics analysis was conducted using bioinformatics analysis, machine learning, and other relevant methods.

The principle of this assay is summarized in Figure 1A. Our discovery cohort comprised samples (HC: BD: SCL: VKH = 48:46:48:48). These diseases encompassed both initial and recurrent states, as well as highly active and hypoactive inflammatory states. The demographic characteristics of the participants are shown in Table 1. Compared with HC, BD exhibited differences in sex distribution. Previous studies have confirmed a higher prevalence of BD in males.^15^ Additionally, the study found that patients in the disease groups had relatively higher body mass index (BMI) values compared to the HC group, particularly in the SCL and VKH groups, with statistically significant differences. However, no studies have described the association between BMI and uveitis or SCL. Therefore, we speculate that the higher BMI may be attributed to changes in the patient’s weight caused by treatment with steroids and biological agents.

**Table 1:** Descriptive statistics of the discovery cohort

Variables	HC (*N* = 48)	BD (*N* = 46)	SCL (*N* = 48)	VKH (*N* = 48)
Gender-no. (%)				
Male	20 (41.7)	29 (71.7)	16 (33.3)	25 (52.1)
Female	28 (58.3)	17 (28.3)	32 (66.7)	23 (47.9)
*p*-value	–	**0.038**^a^	0.399	0.306
Age-year				
Mean ± SD	33.5 ± 8.3	31.0 ± 8.6	38.1 ± 15.6	35.4 ± 11.4
Median (IQR)	34.0 (28.0–40.0)	30.0 (26.0–34.50)	30.0 (26.0–34.50)	34.0 (26.0–44.8)
Range	16.0–50	16.0–59.0	16.0–70.0	16.0–70.0
*p*-value	–	0.163	0.071	0.35
Affected eye-no. (%)				
unilateral	–	8 (17.4)	38 (82.6)	48 (100)
bilateral	–	38 (82.6)	17 (35.4)	0 (0)
Smokeing-no. (%)	18 (37.5)	15 (32.6)	11 (22.9)	11 (22.9)
Drinking-no. (%)	16 (33.3)	9 (19.6)	4 (9.3)	9 (18.8)

HC, healthy control; BD, Behcet’s disease uveitis; SCL, posterior scleritis; VKH, Vogt-Koyanagi-Harada syndrome; SD, standard deviation; IQR, interquartile range. Statistical analyses were performed according to the Mann–Whitney rank test for two-group comparisons.

a Significance was determined by Student’s *t* test for normal data.

### Building plasma proteomic profiles of BD, SCL and VKH

**Figure 2:**
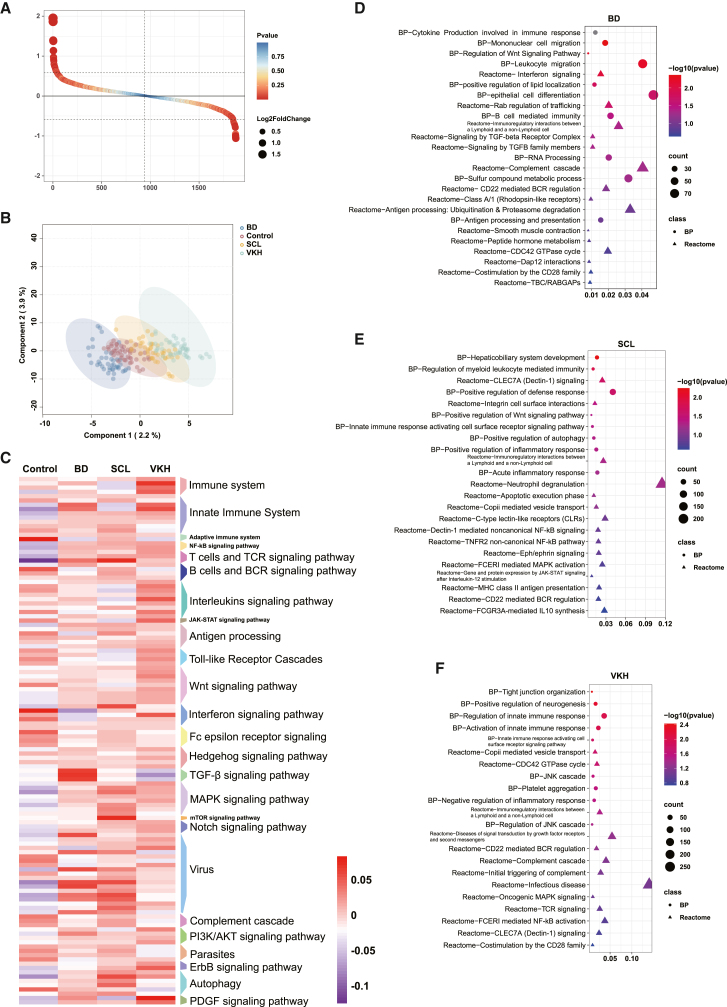
Construction of plasma protein maps for three diseases (A) The scatterplot displays the total number of proteins quantified across four groups. (B) PLS-DA model constructed using 528 DEPs in plasma. (C) Heatmaps displaying the score of pathways obtained through GSVA of the three diseases and HC. Red represents relative activation, while purple represents relative inhibition. (D–F) Bubble plots describing the GSEA results of BD, SCL, and VKH.

To increase the number of identified proteins, we mixed small and large extracellular vesicles from 26 frozen samples with plasma, creating a pre-constructed database consisting of 2432 proteins. Using SWATH-MS technology, we successfully identified 2028 proteins (Figure 2A, Table S1) in 190 plasma samples. PLS-DA analysis using the top 528 DEPs within a 95% confidence interval revealed reliable a discriminative ability among the four groups (Figure 2B).

To provide a more detailed description of the features of these three diseases, we used the expression values of all quantifiable proteins to calculate the average GSVA scores (Figure 2C, Table S2). The heatmap clearly illustrates the suppressed and activated pathways in each disease, and the changes in pathways relative to the HC. Compared to the HC group, we observed significant alterations in the pathways associated with T cells, B cells, Wnt, MAPK, and others during inflammation in the disease groups. Multiple immune pathways were altered in each disease group. For example, the interferon (IFN) signaling pathway and TGF-β signaling pathway were affected in the BD group, the interleukin signaling pathway and Toll-like receptor (TLR) cascades were altered in the SCL group, and the Wnt signaling pathway and TGF-β signaling pathway were changed in the VKH group. The results of the GSVA provided a more vivid depiction of the impact of protein alterations on these diseases and demonstrated the differences and similarities among the four groups.

GSEA further confirmed the enrichment of inflammatory cytokines, Wnt pathway, and the NF-κB pathway in autoimmune uveitis and SCL (Figures 2D–2F, Table S2). Moreover, we found evidence that alterations in the JNK cascade are implicated in the development of VKH. These findings provide new insights into the protein alterations associated with the pathophysiology of different diseases.

### Differential protein profiling between disease and healthy control

**Figure 3:**
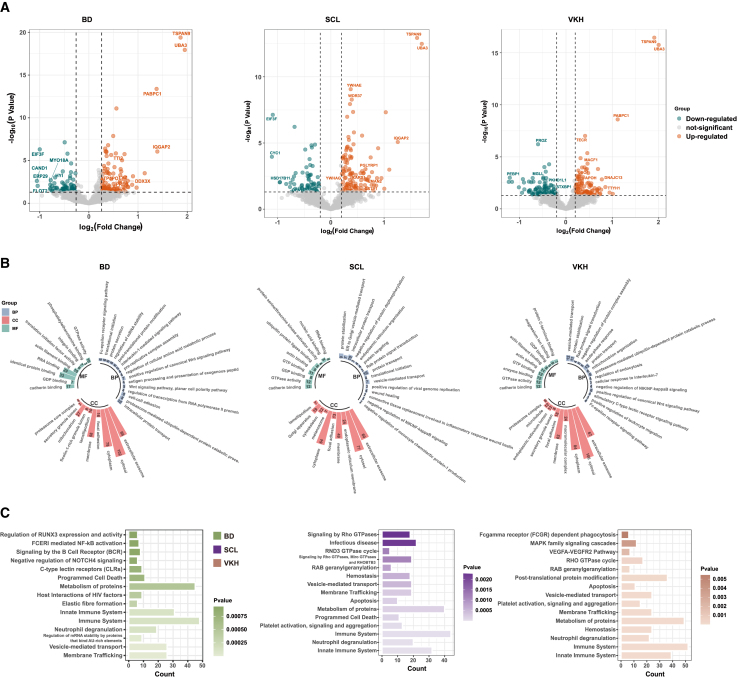
Results of proteomics analysis comparing disease groups with healthy control (A) Volcano plots showing the distribution of DEPs (*p* < 0.05, FC > 1.2 and FC < −1.2). (B) Circular bar chart displaying the GO analysis results. (C) Bar plot showing the pathway enrichment analysis results using Reactome database.

To gain a better understanding of the characteristics of BD, SCL, and VKH compared to HC, we conducted a detailed proteomic analysis to investigate the differences in protein expression between the disease and HC groups. Volcano plots were used to visualize the proteins that exhibited significant differences under disease conditions (Figure 3A, Table S3). By utilizing GO (Figure 3B) and Reactome enrichment analyze (Figure 3C), we gained a more comprehensive understanding of the functional features of proteins that exhibit changes during the disease process compared to HC.

Combining the findings from biological processes and Reactome pathway, we discovered that significant alterations in the regulation of the immune system and neutrophil degranulation across all diseases. C-type lectin receptors, Wnt signaling pathway, and interleukin 1-mediated immune response were enriched in BD. SCL exhibited an abundance of wound healing during the inflammatory response and negative regulation of MCP-1 production. In addition, VKH was enriched in VEGFA-VEGFR2 pathway, MAPK family signaling cascades, and positive regulation of leukocyte migration. These findings revealed altered biological pathways during specific disease processes, that affect the occurrence and progression of the diseases.

### Construction of machine learning models and identification of protein biomarkers

**Figure 4:**
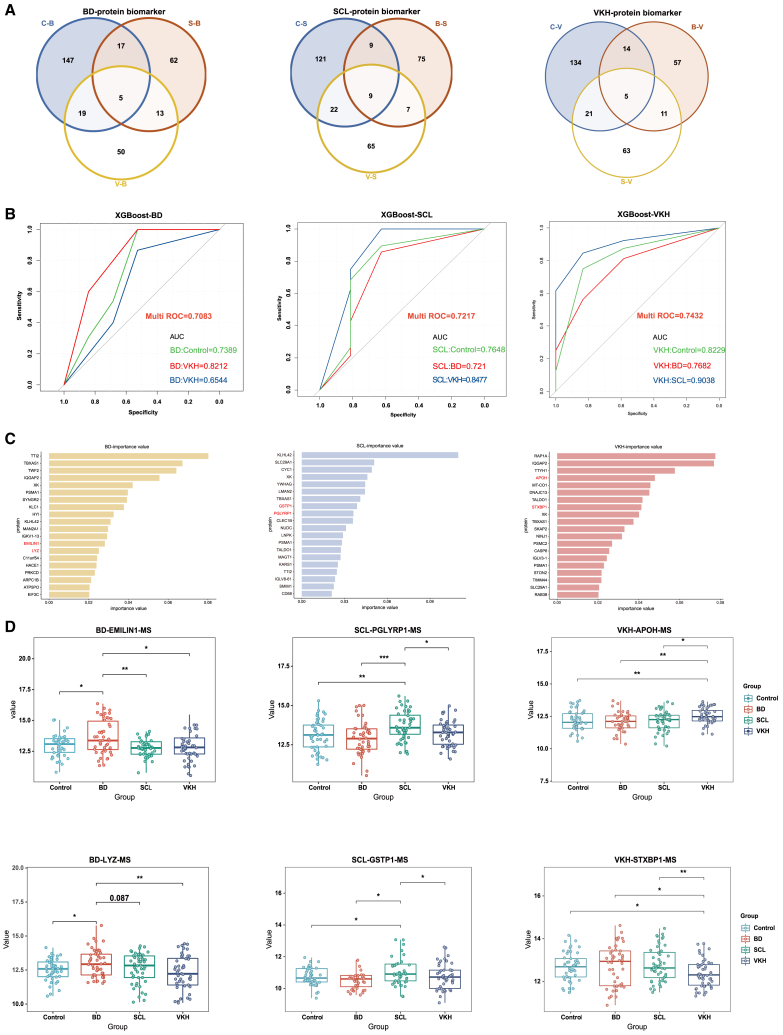
Construction of diagnostic classification models and identification of candidate biomarkers (A) Venn diagrams displaying the DEP overlaps between single disease group and other three groups. (B) ROC curve and AUC values of the XGBoost model. (C) Bar graph showing protein importance scores in the XGBoost model. (D) Protein expression values of candidate biomarkers in plasma. ∗*p* < 0.05, ∗∗*p* < 0.01, ∗∗∗*p* < 0.001, ∗∗∗∗*p* < 0.0001 by one-way ANOVA.

In this study, we developed a machine learning model using the XGBoost algorithm. First, we compared the differences between each individual disease group and the other three groups separately, visualizing them using a Venn diagram (Figure 4A, Table S4). We selected the overlapping proteins in the Venn diagram as a disease-specific protein group for model construction and evaluated the predictive ability of the model using Multi AUC values (Figure 4B). Additionally, we ranked the proteins in the model based on their importance and identified the top twenty most significant proteins (Figure 4C, Table S5). From the top 20 feature proteins, taking into account specificity, function, and expression levels, we selected EMILIN1, LYZ, PGLYRP1, GSTP1, APOH, and STXBP1 for further validation (Figure 4D). Except for LYZ in BD, the other five proteins belonged to the central overlapping region in Figure 4A. The mass spectrometry results showed statistically significant differences among the different groups, confirming the effectiveness of the selected proteins.

### Validation of biomarkers and construction of marker panels

**Figure 5:**
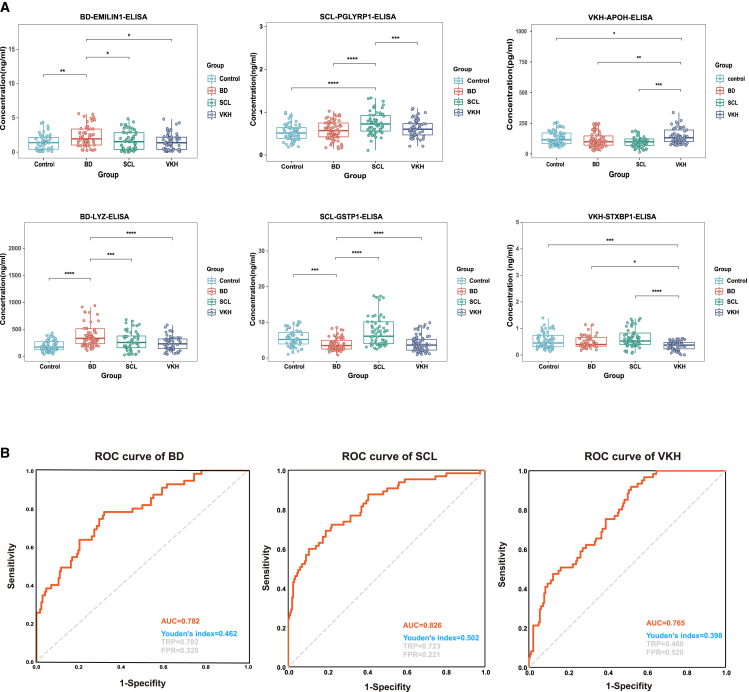
Validation of candidate biomarkers and construction of biomarker panels (A) Concentrations of candidate biomarkers of validation cohort. ∗*p* < 0.05, ∗∗*p* < 0.01, ∗∗∗*p* < 0.001, ∗∗∗∗*p* < 0.0001 by One-Way ANOVA. (B) The AUC of the ROC of potential biomarker panels for the three diseases between a single diseased group versus the other three groups.

The validation cohort consisted 262 individuals, with 65 individuals in each disease group and 67 in the HC group (Table S6). We analyzed 1572 samples using enzyme-linked immunosorbent assay (ELISA) kits, and the validation results of the candidate biomarkers showed significant differences (Figure 5A). To enhance the effectiveness of protein biomarkers for disease diagnosis, we constructed a diagnostic model using the validated candidate biomarkers. By combining the two proteins and conducting ROC curve analysis, we established panels with higher diagnostic efficiency (Figure 5B). The area under the ROC curve for all three panels was greater than 0.75, indicating that these panels could more accurately assess disease risk more accurately and provide more reliable diagnostic results.

**Figure 6:**
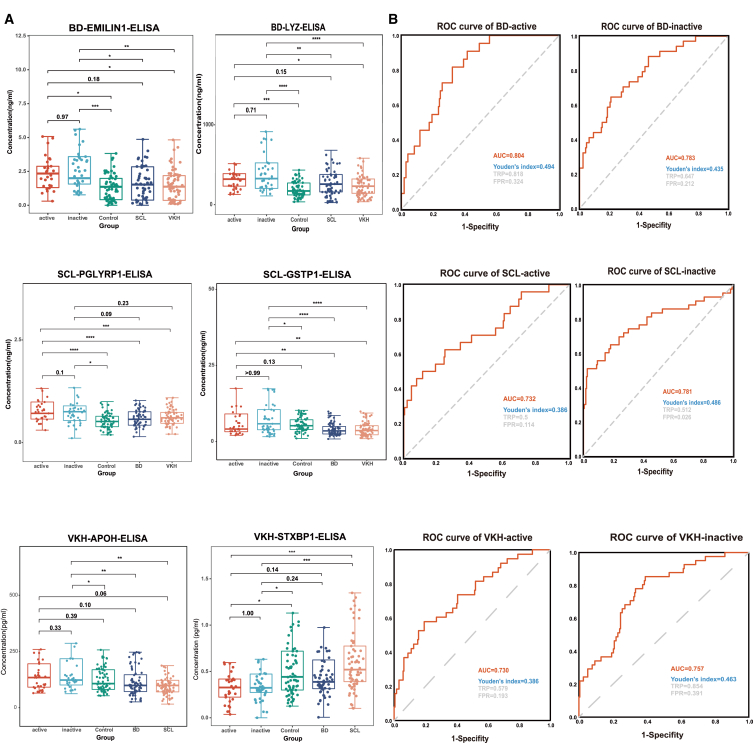
Expression and diagnostic performance of candidate biomarkers at different stages of disease (A) Boxplots showing the expression of candidate biomarkers at different stages of the disease. ∗*p* < 0.05, ∗∗*p* < 0.01, ∗∗∗*p* < 0.001, ∗∗∗∗*p* < 0.0001 by one-way ANOVA. (B) ROC analysis demonstrates the diagnostic performance of biomarker panels at different stages of the disease.

To further investigate the diagnostic ability of candidate biomarkers for different stages of the disease, we evaluated their performance in both active and inactive phases of the disease. The expression levels of these six proteins across various clinical stages of the disease were displayed using boxplots (Figure 6A). Most of the outcomes align well with our expectations, displaying good consistency. Meanwhile, a few results, while maintaining the correct trend, did not exhibit statistically significant differences, which may be due to the limitation of sample size. Subsequently, we conducted a phased ROC analysis on the previously identified biomarker panels. The results showed that the ROC values for all six panels exceeded 0.7 (Figure 6B), indicating that these panels have good diagnostic efficacy across different stages of the disease.

### Predicting potentially treatable drugs

**Figure 7:**
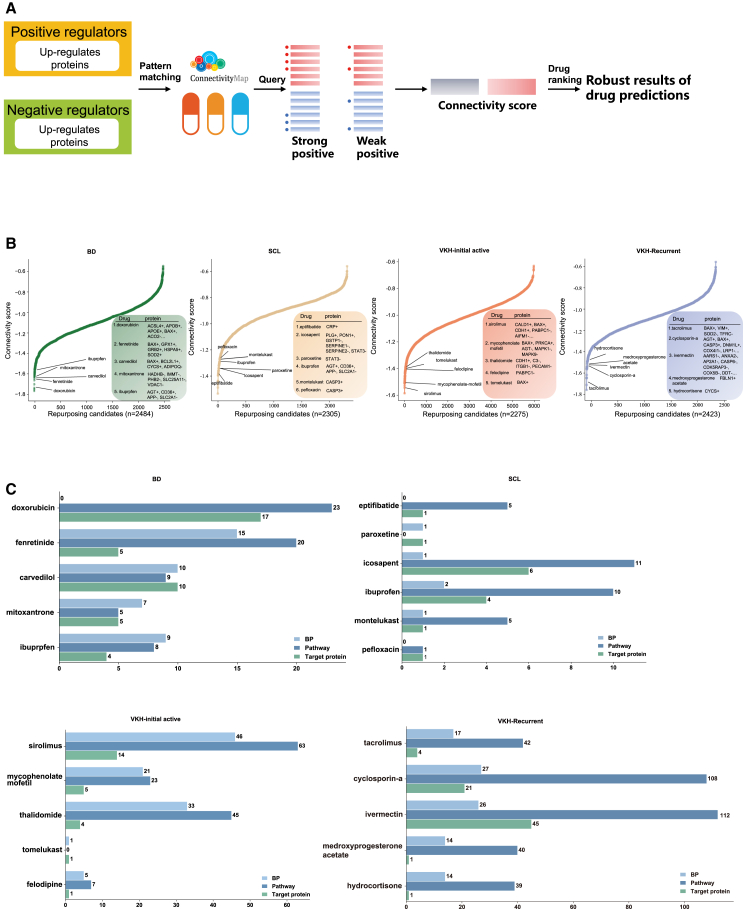
Prediction of potential therapeutic agents (A) Workflow of drug prediction using online tools. (B) Rank plots displaying the ranking of predicted drugs and their negative connectivity scores in BD, SCL, and VKH. The figure highlights the drugs with the most promising clinical application potential, selected from the top 30 drugs. (C) Bar plots depict the count of biological processes, pathways, and target proteins in which drugs are implicated within particular diseases, as derived from the CTD database.

We used the prediction of up-regulated and down-regulated DEPs between disease and healthy control on an online analysis platform to identify small molecule drugs associated with disease treatment (Figure 7A). The drugs were screened using CMap and sorted based on connectivity score.

Potential therapeutic drugs were identified according to the following standards: (1) negative connectivity score; (2) FDA approval or being in phase 3 clinical trials; (3) anti-inflammatory and immunomodulatory effects; (4) targeting proteins on our list and acting in the opposite direction of the protein changes we detected; and (5) significant overlap between the biological pathways implicated by the drugs and those altered in the disease state. The final outcomes included specific drugs for each disease and their corresponding targets (Figure 7B, Table S7). The biological processes and pathways associated with these drugs are shown using bar charts (Figure 7C).

## Discussion

A comprehensive plasma proteomics analysis of BD, VKH, and SCL can help unveil protein changes linked to the onset and progression of these diseases. This approach not only provide valuable insights for diagnosis and treatment but also opens up opportunities for drug discovery. Previous research has revealed that plasma proteomic studies of autoimmune uveitis have primarily focused on either individual diseases or specific stages of multiple diseases, lacking a systematic comparative analysis across all disease stages. In this study, we not only conducted a comprehensive analysis of the entire disease process of BD, SCL, and VKH but also performed separate analyses of the individual stages of these diseases. The objective of our study was to revealed the presence of dysregulated proteins in these three diseases, identified clinically significant biomarkers, and potential therapeutic drugs.

BD is a chronic, recurrent autoimmune disease affecting multiple organ systems. In China, uveitis caused by BD is important cause of blinding eye diseases. Patients with BD often exhibit abnormal and enhanced immune response activation due to immune system dysfunction.^16^ Based on the results of the enrichment analysis, we believe that the ErbB signaling pathway may play a regulatory role in BD. During inflammation, the number of B cells increases, which can promote the production of Amphiregulin.^17^ Amphiregulin is an epidermal growth factor ligand and is expressed by various immune cells during inflammation. Amphiregulin plays key roles in host resistance and tolerance, tissue repair, homeostasis maintenance, local inflammation suppression, and tumor microenvironment immune suppression.^18^ Overexpression of Amphiregulin is associated with autoimmune diseases such as systemic lupus erythematosus,^19^^,^^20^ autoimmune diabetes,^21^ and rheumatoid arthritis.^17^ Additionally, Erb4 is specifically expressed in BD, confirming that the ErbB pathway plays an important role in the development of BD.^22^ The IFN signaling pathway also exhibits specificity for BD. The upregulation of TLR3 during the development of BD can promote the synthesis of interferon-beta (IFN-β), which becomes the predominant type of IFN in BD. IFN-β can trigger the release of monocytes, disrupting immune regulation mechanisms^23^ and leading to immune system imbalance and potential ocular tissue inflammation. Conversely, interferon-gamma (IFN-γ), a member of the IFN family, can activate signal transducer and activator of transcription 1 to initiate the expression of immune effector genes during immune responses. This activation promotes the expression of chemokines, cytokines, and antigen presentation molecules. IFN-γ can also stimulate the expression of TLRs to promote the activation of NF-κB and macrophages.^24^ In summary, it can be speculated that ErbB and IFN signaling pathways play important roles in the development of BD by promoting the production and activation of immune regulatory factors. EMILIN1 is an extracellular matrix glycoprotein associated with elastic fibers and is believed to possess anti-proliferative effects.^25^^,^^26^ It is involved in the negative regulation of macrophage migration and transforming growth factor signaling pathways. When the expression level of EMILIN1 increases, it can inhibit the migration of macrophages to areas of inflammation, as well as the production and release of inflammatory factors, such as TNF-α and IL-1β. This, in turn, helps regulate the immune response and the progression of the inflammatory process in BD. LYZ, an antimicrobial protein present in neutrophils and macrophages, primarily functions to hydrolyze peptidoglycans. LYZ levels in the plasma of patients with BD are relatively high.^27^ LYZ can promote the aggregation of macrophages and the activation of inflammasomes by hydrolyzing bacterial peptidoglycans, exhibiting both antibacterial and immune-regulatory activities. Therefore, EMILIN1 and LYZ may be closely related to the pathogenesis of BD and the regulation of infection and inflammation, potentially playing positive and active anti-inflammatory roles.

Most cases of SCL are caused by immune system disorders, and it is relatively rare in patients with scleritis. Enrichment analysis showed that neutrophil degranulation, interleukin signaling pathways, and C-type lectin-like receptors (CLRs) play important roles in the development of SCL. Neutrophils are important inflammatory cells that originate from white blood cells in the bone marrow. When inflammation occurs, they migrate to sites of tissue inflammation via chemotactic signals and induce inflammation through a receptor-mediated respiratory burst and degranulation.^28^ In uveitis, cathelicidin released from neutrophils not only induces the differentiation of Th17 cells, but also promotes their survival,^29^^,^^30^ thereby promoting inflammation. Additionally, studies have shown that IL-17 exacerbates inflammatory responses in a mouse model of uveitis by inducing neutrophil differentiation. Similarly, after stimulation with IL-6 and IL-23, neutrophils autonomously produce IL-17.^31^ Therefore, it is reasonable to speculate that neutrophil degranulation may activate interleukin signaling pathways and CLRs during the development of SCL. This could lead to the release of inflammatory mediators and enzymes which, in turn, trigger inflammation and edema in scleral tissues. PGLYRP1 is a member of the PGRP-S protein family and is considered an active regulator of the immune response.^32^ It participates in the negative regulation of inflammatory responses, natural killer cell differentiation, and biological processes involving type II IFNs. In the SCL group, PGLYRP1 and GSTP1 were significantly overexpressed compared to other groups. In arthritis, PGLYRP1 exerts anti-inflammatory effects by reducing the levels of pro-inflammatory cytokines and chemokines in the blood.^33^ Therefore, increased levels of PGLYRP1 may play an anti-inflammatory role in the development of SCL, helping to alleviate inflammatory responses and associated symptoms. GSTP1 plays an important role in the clearance of oxidative stress (reactive oxygen species (ROS)),^34^ and inhibiting ROS therapy can reduce the levels of ROS, inflammatory mediators, and NF-κB in autoimmune uveitis,^35^ thus inhibiting inflammation. Therefore, it is reasonable to speculate that GSTP1 protects ocular tissues in BD by reducing oxidative stress and regulating the release of inflammatory mediators. Through proteomic analysis, we found that PGLYRP1 and GSTP1 are closely related to the pathogenesis and inflammatory regulation of SCL. These findings provide clues for potential etiological mechanisms and therapeutic targets for SCL.

VKH is a blinding eye disease caused by immune system disorders that attack melanocytes and manifest as changes in the skin and hair, abnormal hearing, and central nervous system involvement. It is often misdiagnosed, leading to recurrent inflammation and, potentially, subsequent blindness.^36^ Previous studies have mainly focused on analyzing the active phase of VKH inflammation. In this study, we included patient samples from all stages, such as initial onset, relapse, high activity, and low activity, to provide a more comprehensive analysis. Our enrichment results suggested that the TGF-β signaling pathway, complement cascade, and PDGF signaling pathway were involved in the occurrence of inflammatory responses. TGF-β is an immune regulatory factor that plays a crucial role in regulating and propagating autoimmune diseases. It mainly induces the production of antigen-specific tolerance in inactivated naive T-cells by regulating cytokine production. In addition, the production of TGF-β is associated with Th3 regulatory T cells, and the increase in Th3 regulatory T cells helps maintain immune homeostasis, thereby inhibiting the development of autoimmune diseases.^37^ Therefore, we can speculate that the downregulation of the TGF-β signaling pathway promotes the progression of VKH inflammation by reducing the secretion of Th3 regulatory cells. Compared with the other three groups, the PDGF signaling pathway was significantly upregulated in the VKH group. Previous studies have found that the PDGF signaling pathway is activated and expressed in the plasma of patients with VKH, although the exact cause remains unknown,^38^ and its specific functional role remains unclear. In VKH group, APOH and STXBP1 were the DEPs with high and low expression, respectively. APOH, also known as Beta-2-glycoprotein, is associated with high-density lipoproteins (HDL). It binds mainly to HDL in the plasma and plays a key role in lipid metabolism and transport. The main biological processes involving APOH include the clearance of apoptotic bodies and liposomes, as well as interfering with the protein C pathway.^39^ Currently, no research has suggested a link between APOH and VKH; however, APOH can present antigens to T cell subsets^40^ and exhibit anti-inflammatory effects through complement binding during inflammatory reactions and immune processes.^41^ Therefore, we speculate that in VKH, the increased expression of APOH may enhance the recognition and response of the immune system to specific antigens by presenting antigens to T cell subsets, leading to excessive immune responses. APOH may also bind to the complement system to enhance its anti-inflammatory capabilities and mitigate the inflammatory response in VKH. STXBP1 is a protein involved in vesicle transport and neurotransmitter release, as well as cellular response to type II IFNs and the release of granules from mast cells. Its correlation with immune cells, such as CD4^+^ T cells, macrophages, and dendritic cells,^42^ suggests that the STXBP1 gene may play a role in regulating immune response processes. Additionally, the STXBP1/STX1 axis is believed to be closely associated with the cytotoxic activity of NK and CD8^+^ T cells, potentially accounting for approximately 50% of their cytotoxic activity.^43^ In VKH, impaired functionality of this axis may affect the cytotoxic activity of NK and CD8^+^ T cells, preventing them from correctly identifying and eliminating abnormal cells and potentially leading to excessive or insufficient autoimmune reactions. These hypotheses require further experimental validation and a deeper understanding of the impact of APOH and STXBP1 on immune and inflammatory mechanisms in VKH. The presence of these proteins was verified by ELISA, which indicated that changes in STXBP1 and APOH were specifically associated with VKH.

Building on these findings, drug screening is based on the principle that the most effective drugs can reverse the molecular characteristics a disease with minimal side effects. We used the L1000-based CMap for drug repurposing analysis. The L1000 is a low-cost, high-throughput transcriptome analysis method with high reproducibility.^44^ CMap can link diseases with drugs through proteins, and screen for drugs that can be repurposed by evaluating the connectivity of disease-drug relationships. By considering drug function, target proteins, and connectivity scores, we ultimately identified valuable drugs for each disease. They primarily belong to the categories of glucocorticoids, leukotriene receptor antagonists, and platelet aggregation inhibitors. Furthermore, through literature review, we have discovered that these drugs possess certain functions in anti-inflammation and immune regulation. This suggests that these drugs may achieve therapeutic purposes by modulating the immune and anti-inflammatory pathways involved in the disease. The drugs we have selected are currently in clinical phase III trials or have already been approved by the FDA. This indicates that these drugs have undergone relatively thorough validation in terms of safety and effectiveness, and they hold promising clinical application prospects in the treatment of BD, SCL, and VKH.

Compared to other biological samples, plasma exhibits significant advantages in the study of ophthalmic proteomics, hence attracting a growing number of research endeavors. Firstly, plasma is rich in protein types, which play vital roles in various physiological and pathological processes of the body, enabling timely reflection of the body’s physiological and pathological states. Secondly, compared to other biological samples, collecting plasma samples is relatively simple with minimal trauma, imposing less physical burden on patients. This facilitates more convenient sample collection from large-scale populations, contributing to large-cohort proteomic studies. Thirdly, plasma samples are generally stable, making them easy to preserve and transport. In the future, these biomarkers still need to be validated in a larger population to determine their sensitivity, specificity, and diagnostic accuracy before clinical application. Additionally, suitable clinical detection methods need to be developed.

### Limitations of the study

Although we conducted detailed and in-depth analyses of different disease stages, there are certain limitations to our research. First, our comparative analysis mainly focused on four groups (HC, BD, SCL, and VKH), which limited the scope of our research results and did not include comparison with other types of uveitis. Second, although we obtained some preliminary results, further verification in a larger cohort is needed to confirm the applicability of these results in clinical practice. Therefore, future research should aim to address these limitations to improve our understanding of pathogenesis of the disease and apply biomarkers more effectively in clinical practice.

## Resource availability

### Lead contact

For further information on reagents and resources, please contact the lead contact, X.Z. (e-mail: xzhang08@tmu.edu.cn).

### Materials availability

This study did not generate novel reagents.

### Data and code availability

• The proteomics data obtained through mass spectrometry have been deposited at obtained through mass spectrometry and are publicly available as of the date of publication. Accession numbers are listed in the key resources table.
• This study did not report any original code.
• Any additional information required to reanalyze the data reported in this paper is available from the lead contact upon request.

## Acknowledgments

This research was supported by 10.13039/501100001809National Natural Science Foundation of China, China (82371044, 82171042) and Tianjin Key Medical Discipline (Specialty) Project, China (TJWJ2023XK009). 10.13039/501100010104Tianjin Medical University “Clinical Talent Training 123 Climbing Plan”, China. Tianjin Binhai New Area Health Research Project, China (Grant 2023BWKQ018).

## Author contributions

Conceptualization: X.Z. and X.L.; Collected and handled the clinical samples: X.L., L.W., and J.A.; MS data analysis and visualization: X.L., A.J., and L.W.; ELISA validation: X.L., Q.T., and H.Z.; Project supervision: X.Z. and X.L.; Writing – Original Draft: X.L., A.J., and X.Z.; Writing – Review and Editing: all authors.

## Declaration of interests

X.Z., X.L., and A.J. are the inventor of the patent CN202410077768.2.

X.Z., X.L., A.J., and L.W. are the inventors of the patent CN202410077698.0.

X.Z., X.L., L.W., and A.J. are the inventors of the patent CN202410077616.2.

## STAR★Methods

### Key resources table

**Table:** 

REAGENT or RESOURCE	SOURCE	IDENTIFIER
**Biological samples**		

Plasma samples from patients	Tianjin Medical University Eye Hospital	This paper

**Chemicals, peptides, and recombinant proteins**		

Human APOH ELISA Kit	FineTest	Cat#EH0531
Human GSTP1 ELISA Kit	FineTest	Cat#EH1233
Human STXBP1 ELISA Kit	FineTest	Cat#EH12697
Human EMILIN1 ELISA Kit	FineTest	Cat#EH2997
Human PGLYRP1 ELISA Kit	FineTest	Cat#EH3565
Human LZMC ELISA Kit	FineTest	Cat#EH4052
Amicon® Ultra Centrifugal Filters	Merck	Cat#UFC501096
Protease inhibitor	Roche	Cat#4906837001
Trypsin	Promega	Cat#V5117
Urea	Sigma	Cat#U4884-500G
Dithiothreitol	Sigma	Cat#0632-100G
Iodoacetamide	Sigma	Cat#I1149-25G
NH_4_HCO_3_	Sigma Aldrich, Germany	BioUltra, >99.5%
Triton lysate buffer	Solarbio	Cat#T8200

**Critical commercial assays**		

Liquid Chromatograph	AB SCIEX	Eksigent nanoLC 415
Mass spectrometer	AB SCIEX	Triple TOF 6600

**Deposited data**		

Mass spectrometry data	ProteomeXchange	PXD052546

**Software and algorithms**		

R (v4.3.0)	R Project	https://www.r-project.org/
SPSS Statistic 26.0	IBM	https://www.ibm.com/cn-zh
UniProt	UniProt Consortium	https://www.uniprot.org/
PeakView v2.2	AB SCIEX	N/A
ProteinPilot software v5.0.1	AB SCIEX	N/A
GSEA	Open source	https://www.gsea-msigdb.org/
Hiplot Pro	N/A	https://hiplot.com.cn
Bioinformatics	N/A	https://www.bioinformatics.com.cn
Fig Draw	N/A	https://www.figdraw.com/
DAVID	N/A	https://david.ncifcrf.gov/
Connectivity Map	N/A	https://clue.io/

### Experimental model and study participant details

#### Clinical samples

All participants in this study were treated at the Tianjin Medical University Eye Hospital between January 2018 and December 2022. Including HC (n=48, 39.3±10.3, male:20, female:28), BD (n=46, 31.0±8.6, male:29, female:17), SCL (n=48, 38.1±15.6, male:16, female:32) and VKH (n=48, 35.4±11.4, male:25, female:23). BD, VKH, and SCL were diagnosed by professional ophthalmologists and the inflammation level were evaluated using the standardized uveitis nomenclature.^45^

Informed consent was obtained from all patients included in the study. All procedures adhered to the Declaration of Helsinki, and ethical approval was obtained from the Ethics Committee of the Tianjin Medical University Eye Hospital, Tianjin, China (approval number: 2021KY(L)-45).

#### Inclusion and diagnostic criteria

The inclusion criteria included: a. Patients with autoimmune uveitis (including BD, VKH), as well as SCL; b. Aged between 16 and 70 years old; c. Free from diabetes, hypertension, hyperlipidemia, cardiovascular diseases, mental illnesses, and other systemic organic diseases; d. No history of perforating injury to the eyeball or intraocular surgery; no other ocular lesions except cataract in both eyes.

BD was diagnosed in strict accordance with the diagnostic criteria established by the International Study Group for Behçet’s Disease.^46^ For VKH patients, the primary diagnostic features centered on bilateral diffuse choroiditis, serous retinal detachment, and varying degrees of inflammatory cell infiltration.^47^ Meanwhile, SCL was primarily diagnosed based on the diagnostic criteria proposed by Mccluskey^12^ and Cheung.^11^

### Method details

#### Plasma collection

Fresh venous blood samples from all phases of BD, SCL, and VKH were collected and stored in EDTA tubes. After standing for more than 30 minutes, the samples were centrifuged at 4°C and 1800g for 15 minutes to obtain plasma. The plasma was then stored at -80°C for future experimental use.

#### Sample preparation before mass spectrometry detection

2μl plasma samples were resuspended in 8M urea lysis buffer (8M urea, 1mM NaF, 1mM Na_3_VO_4_, 50mM NH_4_HCO_3_, and 1× complete protease inhibitor mixture) containing protease inhibitor (Roche, Switzerland). An equal number of samples were reduced with 1μl of dithiothreitol (final concentration 10mM) and alkylated with 4μl of iodoacetamide (final concentration 40mM). After reduction and alkylation, the sample was loaded onto a Amicon® Ultra Centrifugal Filters (Merck, Germany) and digested with trypsin (Promega, USA) at a 50:1 protein-to-enzyme ratio for 12h at 37°C. Washing with 50mM NH_4_HCO_3_ (> 99.5%, Sigma-Aldrich) in order to obtain tryptic peptides, and terminating the digestion via 1% (v/v) FA (LiChroPUR, 98–100%, Merck). The peptides were desiccated accessing SpeedVac (Thermo Scientific, USA).

#### Construction of spectral library by information-dependent acquisition mode

We generated a Theoretical fragment ions-mass spectrometry (SWATH-MS) reference spectral library to quantify proteins in plasma. Specific library construction method as previously reported by Wu et al.^48^ The proteomic analysis was carried out using an Eksigent nano-LC 415 (AB SCIEX, Framingham, MA, USA) coupled with a Triple TOF 6600 mass spectrometer (AB SCIEX, Framingham, MA, USA). The peptides were dissolved in mobile phase A solvent (0.1% formic acid, 2% ACN, 97.9% water) and loaded onto an AB SCIEX trap column (10 × 0.3mm; C18 packing specification is 5μm, 120Å) at a speed of 10μl/min. Phase B solvent (97.9% acetonitrile, 2% water, 0.1% formic acid) was used to elute the trap column at a flow rate of 5μl per minute. The eluted peptide segments were subjected to an analytical column (150 × 0.3mm; C18 packing specification is 3μm, 120Å) and electrospraying. The peptides were resolved over a 60 minutes nano-LC gradient (linear gradient from 5 to 80% LC buffer B [0.1% FA and 2% water in acetonitrile] over 55 minutes and then to 5% buffer B over 5 minutes). The TOF-MS accumulation time was set as 0.25s with a mass range of 300-1500Da. The charge state was set from +2 to +5. The mass tolerance was less than 50ppm, and 60 candidate ions were submitted for each cycle.

#### SWATH-MS for quantitative proteomics

For SWATH-MS, the same chromatographic parameters described above were used in the IDA model for ion library construction. The accumulation time of TOF-MS was set at 0.25s, and the high-sensitivity scanning mode was used for MS/MS. Furthermore, 90 variable windows with 36ms accumulation time were used based on the SWATH (Sequential Window Acquisition of all Theoretical fragment ions) variable window calculator V1.1. The scanning mass range was 100-1500Da. Typically, 1μg peptides for plasma were injected for analysis.

#### Machine learning

A XGBoost model was implemented by the R package “xgboost”, the whole datasets were randomly divided into a training group and a testing group at a ratio of 7:3. The “xgb.instance” packages was used to rank proteins according their importance, and the “pROC” package was used to constructed the receiver operating characteristic (ROC) curves.

#### Validation by enzyme linked immunosorbent assay

Beta-2-glycoprotein 1 (APOH, FineTest, China), Syntaxin-binding protein 1 (STXBP1, FineTest, China), Peptidoglycan recognition protein 1 (PGLYRP1, FineTest, China), Glutathione S-transferase P (GSTP1, FineTest, China), Elastin microfibril interface-located protein 1 (EMILIN1, FineTest, China), Lysozyme C (LYZ, FineTest, China) were carried out in accordance with the instructions. 100μl standard or plasma dilutions were incubated 90 minutes. Then, 100μl of antibody working solution was added and incubated for 60 minutes. After washing, HRP-Streptavidin Conjugate solution was added and incubated for 30 minutes. Tetramethylbenzidine solution was added to each well and incubated for 10-20 minutes. Finally, the reaction was interrupted by a stop solution. The optical density values were measured at 450nm (referenced to 540nm) using Tecan Infinite 200 Pro plate reader (Zurich, Switzerland).

#### Prediction of potential therapeutic drugs through connectivity map

Given that the three groups of samples covered multiple stages of the disease, each with varying characteristics, we conducted drug prediction for the different stages of the disease. Potential therapeutic agents were available by inputting DEPs obtained from diseases into a Connectivity Map (CMap, https://clue.io/). The CMap based L1000 assay system using up-regulated and down-regulated proteins to identify potential drug candidates. The drugs were screened, followed by a detailed analysis of the candidates via DrugBank (https://go.drugbank.com/) and PubChem (https://pubchem.ncbi.nlm.nih.gov/). Drug-disease-associated biological processes, pathways and protein targets were acquired from the Comparative Toxicogenomics Database (CTD, http://ctd.mdibl.org).

### Quantification and statistical analysis

#### Data analysis and statistical method

Using ProteinPilot software (v5.0.1; AB SCIEX), IDA data, reference library construction, and protein identification were analyzed using the UniProt human SwissProt database (https://www.uniprot.org/, released July 2019). The SWATH quantitation microapp of PeakView Software (v2.2, AB SCIEX) was used to the extract SWATH-MS peaks. The entire process took 12 minutes and the detailed parameters were as follows: six peptides and six transitions, shared and modified peptides excluded, peptide confidence > 99%, XIC width 50ppm, and FDR < 1%. Quantitative values were based on peak areas. Post-processing of quantitative data included log_2_ transformation and median normalization using the “preprocessCore” package in R. Differential analysis was performed using the “stats” package in R. Differentially expressed proteins (DEPs) were screened p-values < 0.05 and fold change > 1.2. SPSS software (v26.0) was used to analyze statistical significance. Student’s t-test was used to analyze data with normal distribution and uniform variance; otherwise, the Mann-Whitney rank test and One-way ANOVA were applied. All data are expressed as mean ± standard deviation. Significant differences are indicated by asterisks: ∗p < 0.05, ∗∗p < 0.01, ∗∗∗p < 0.001, and ∗∗∗∗p < 0.0001. A binary logistic regression algorithm was used to analyze multivariate ROC curves. ROC curve analysis was performed using SPSS to evaluate sensitivity, specificity, and predictive value.

#### Bioinformatics analysis and feature selection

Veen plots, bar plots, volcano plots, heatmap, bubble charts, circular bar charts, rank plots with labels and boxplots were performed by the online biomedical visualization platform Hiplot Pro (https://hiplot.com.cn) and Bioinformatics (http://www.bioinformatics.com.cn/). Graphical materials were sourced from the Fig Draw online website (https://www.figdraw.com/). The acquired images were adjusted using Adobe Illustrator (Adobe, USA). GSEA software were used to perform gene set enrichment analysis (GSEA), and gene set variation analysis (GSVA) were conducted by “clusterProfiler” packages in R. Gene Ontology (GO) enrichment analysis and Reactome pathway analysis were performed through the bioinformatics resource platform DAVID (https://david.ncifcrf.gov/). Partial least square discriminant analysis (PLS-DA) was established by MetaboAnalys v6.0 (https://www.metaboanalyst.ca/).

## Supplemental information

Table S1. Identified proteins in plasma via SWATH-MS, related to Figure2A

Table S2. Pathway of GSVA and GSEA for three diseases, related to Figure 2C-F

Table S3. Differentially expressed proteins between the BD/SCL/VKH and healthy control group in plasma, related to Figure 3A

Table S4. Results of Venn diagram analysis among four groups across three diseases, related to Figure 4A

Table S5. Importance ranking of proteins in XGBoost model, related to Figure 4C

Table S6. Clinical information of the discovery cohort, related to Figure 5A

Table S7. Identification of potential drugs for three diseases, related to Figure 7B

## References

[1] Zeboulon N., Dougados M., Gossec L. (2008). Prevalence and characteristics of uveitis in the spondyloarthropathies: a systematic literature review. Ann. Rheum. Dis..

[2] Dick AD T.N., Sorg R., Zhao C., Chao J., Joshi A., Skup M. (2016). Risk of Ocular Complications in Patients with Noninfectious Intermediate Uveitis, Posterior Uveitis, or Panuveitis. Ophthalmology.

[3] Yang P., Zhong Z., Du L., Li F., Chen Z., Zhu Y., Zhang W., Huang F., Ye X., Su G., Kijlstra A. (2021). Prevalence and clinical features of systemic diseases in Chinese patients with uveitis. Br. J. Ophthalmol..

[4] P Y. (2021).

[5] Greco A., De Virgilio A., Ralli M., Ciofalo A., Mancini P., Attanasio G., de Vincentiis M., Lambiase A. (2018). Behçet's disease: New insights into pathophysiology, clinical features and treatment options. Autoimmun. Rev..

[6] Ye Z., Deng B., Wang C., Zhang D., Kijlstra A., Yang P. (2016). Decreased B and T lymphocyte attenuator in Behcet's disease may trigger abnormal Th17 and Th1 immune responses. Sci. Rep..

[7] Yang P., Zhang Z., Zhou H., Li B., Huang X., Gao Y., Zhu L., Ren Y., Klooster J., Kijlstra A. (2005). Clinical Patterns and Characteristics of Uveitis in a Tertiary Center for Uveitis in China. Curr. Eye Res..

[8] Liu H., Zhang P., Li F., Xiao X., Zhang Y., Li N., Du L., Yang P. (2023). Identification of the immune-related biomarkers in Behcet’s disease by plasma proteomic analysis. Arthritis Res. Ther..

[9] Yang P., Ren Y., Li B., Fang W., Meng Q., Kijlstra A. (2007). Clinical Characteristics of Vogt–Koyanagi–Harada Syndrome in Chinese Patients. Ophthalmology.

[10] Luo K., Cai H., Hu Y., Jin C., Gan X., Deng Y., Lu M., Chen H., Su L., Chen G., Chi W. (2021). Distinguishing Microvasculature Features of Vogt-Koyanagi-Harada in Patients in Acute and Convalescent Phases Using Optical Coherence Tomography Angiography. Ocul. Immunol. Inflamm..

[11] Cheung C.M.G., Chee S.-P. (2012). Posterior Scleritis in Children: Clinical Features and Treatment. Ophthalmology.

[12] McCluskey P.J., Watson P.G., Lightman S., Haybittle J., Restori M., Branley M. (1999). Posterior scleritis. Ophthalmology.

[13] Zhong W., Edfors F., Gummesson A., Bergström G., Fagerberg L., Uhlén M. (2021). Next generation plasma proteome profiling to monitor health and disease. Nat. Commun..

[14] Orlando E., Aebersold R. (2019). On the contribution of mass spectrometry-based platforms to the field of personalized oncology. TrAC, Trends Anal. Chem..

[15] Yang P., Fang W., Meng Q., Ren Y., Xing L., Kijlstra A. (2008). Clinical Features of Chinese Patients with Behçet’s Disease. Ophthalmology.

[16] Tong B., Liu X., Xiao J., Su G. (2019). Immunopathogenesis of Behcet's Disease. Front. Immunol..

[17] Mahendra A., Yang X., Abnouf S., Adolacion J.R.T., Park D., Soomro S., Roszik J., Coarfa C., Romain G., Wanzeck K. (2019). Beyond Autoantibodies: Biologic Roles of Human Autoreactive B Cells in Rheumatoid Arthritis Revealed by RNA-Sequencing. Arthritis Rheumatol..

[18] Zaiss D.M.W., Gause W.C., Osborne L.C., Artis D. (2015). Emerging functions of amphiregulin in orchestrating immunity, inflammation, and tissue repair. Immunity.

[19] Ishii T., Onda H., Tanigawa A., Ohshima S., Fujiwara H., Mima T., Katada Y., Deguchi H., Suemura M., Miyake T. (2005). Isolation and expression profiling of genes upregulated in the peripheral blood cells of systemic lupus erythematosus patients. DNA Res..

[20] Melderis S., Warkotsch M.T., Dang J., Hagenstein J., Ehnold L.I., Herrnstadt G.R., Niehus C.B., Feindt F.C., Kylies D., Puelles V.G. (2022). The Amphiregulin/EGFR axis protects from lupus nephritis via downregulation of pathogenic CD4(+) T helper cell responses. J. Autoimmun..

[21] Raugh A., Jing Y., Bettini M.L., Bettini M. (2023). The amphiregulin/EGFR axis has limited contribution in controlling autoimmune diabetes. Sci. Rep..

[22] Xavier J.M., Krug T., Davatchi F., Shahram F., Fonseca B.V., Jesus G., Barcelos F., Vedes J., Salgado M., Abdollahi B.S. (2013). Gene expression profiling and association studies implicate the neuregulin signaling pathway in Behcet's disease susceptibility. J. Mol. Med..

[23] Lee M.T., Hooper L.C., Kump L., Hayashi K., Nussenblatt R., Hooks J.J., Detrick B. (2007). Interferon-beta and adhesion molecules (E-selectin and s-intracellular adhesion molecule-1) are detected in sera from patients with retinal vasculitis and are induced in retinal vascular endothelial cells by Toll-like receptor 3 signalling. Clin. Exp. Immunol..

[24] Hu X., Ivashkiv L.B. (2009). Cross-regulation of signaling pathways by interferon-gamma: implications for immune responses and autoimmune diseases. Immunity.

[25] Modica T.M.E., Maiorani O., Sartori G., Pivetta E., Doliana R., Capuano A., Colombatti A., Spessotto P. (2017). The extracellular matrix protein EMILIN1 silences the RAS-ERK pathway via α4β1 integrin and decreases tumor cell growth. Oncotarget.

[26] Harris S.E., Riggio V., Evenden L., Gilchrist T., McCafferty S., Murphy L., Wrobel N., Taylor A.M., Corley J., Pattie A. (2017). Age-related gene expression changes, and transcriptome wide association study of physical and cognitive aging traits, in the Lothian Birth Cohort 1936. Aging (Albany NY).

[27] Latorre J., Lluch A., Ortega F.J., Gavaldà-Navarro A., Comas F., Morón-Ros S., Rodríguez A., Becerril S., Villarroya F., Frühbeck G. (2021). Adipose tissue knockdown of lysozyme reduces local inflammation and improves adipogenesis in high-fat diet-fed mice. Pharmacol. Res..

[28] Agrawal R L.A., Restori M., Pavesio C., Sagoo M.S. (2006). NODULAR POSTERIOR SCLERITIS: Clinico-Sonographic Characteristics and Proposed Diagnostic Criteria. Retina.

[29] Zheng J., Wang Y., Hu J. (2023). Study of the shared gene signatures of polyarticular juvenile idiopathic arthritis and autoimmune uveitis. Front. Immunol..

[30] Minns D., Smith K.J., Alessandrini V., Hardisty G., Melrose L., Jackson-Jones L., MacDonald A.S., Davidson D.J., Gwyer Findlay E. (2021). The neutrophil antimicrobial peptide cathelicidin promotes Th17 differentiation. Nat. Commun..

[31] Taylor P.R., Roy S., Leal S.M., Sun Y., Howell S.J., Cobb B.A., Li X., Pearlman E. (2014). Activation of neutrophils by autocrine IL-17A-IL-17RC interactions during fungal infection is regulated by IL-6, IL-23, RORgammat and dectin-2. Nat. Immunol..

[32] Yashin D.V., Sashchenko L.P., Georgiev G.P. (2021). Mechanisms of Action of the PGLYRP1/Tag7 Protein in Innate and Acquired Immunity. Acta Naturae.

[33] Telegin G.B., Chernov A.S., Minakov A.N., Balmasova I.P., Romanova E.A., Sharapova T.N., Sashchenko L.P., Yashin D.V. (2022). Short Peptides of Innate Immunity Protein Tag7 Inhibit the Production of Cytokines in CFA-Induced Arthritis. Int. J. Mol. Sci..

[34] Salimi S., Nakhaee A., Jafari M., Jahantigh D., Sandooghi M., Zakeri Z., Shahrakipour M., Naghavi A., Farajian-Mashhadi F. (2015). Combination Effect of GSTM1, GSTT1 and GSTP1 Polymorphisms and Risk of Systemic Lupus Erythematosus. Iran. J. Public Health.

[35] Hsu S.M., Yang C.H., Teng Y.T., Tsai H.Y., Lin C.Y., Lin C.J., Shieh C.C., Chen S.H. (2020). Suppression of the Reactive Oxygen Response Alleviates Experimental Autoimmune Uveitis in Mice. Int. J. Mol. Sci..

[36] Du L., Kijlstra A., Yang P. (2016). Vogt-Koyanagi-Harada disease: Novel insights into pathophysiology, diagnosis and treatment. Prog. Retin. Eye Res..

[37] Thorbecke G.J., Umetsu D.T., deKruyff R.H., Hansen G., Chen L.Z., Hochwald G.M. (2000). When engineered to produce latent TGF-beta1, antigen specific T cells down regulate Th1 cell-mediated autoimmune and Th2 cell-mediated allergic inflammatory processes. Cytokine Growth Factor Rev..

[38] Bonacini M., Cimino L., De Simone L., Bolletta E., Gozzi F., Soriano A., Muratore F., Zerbini A., Fontana L., Salvarani C., Croci S. (2021). Vogt-Koyanagi-Harada patients show higher frequencies of circulating NKG2D(pos) NK and NK T cells. Clin. Exp. Immunol..

[39] Irman Š., Škarabot M., Muševič I., Božič B., Božič B. (2011). Thrombomodulatory Effect of Anti-B2-Glycoprotein I Antibodies on Crystalline Annexin A5 on Phospholipid Bilayers, as Observed by Atomic Force Microscopy. EJIFCC.

[40] Champagne E., Martinez L.O., Vantourout P., Collet X., Barbaras R. (2005). Role of apolipoproteins in gammadelta and NKT cell-mediated innate immunity. Immunol. Res..

[41] Niessen H.W., Lagrand W.K., Rensink H.J., Meijer C.J., Aarden L., Hack C.E., Visser C. (2000). Apolipoprotein H, a new mediator in the inflammatory changes ensuring in jeopardised human myocardium. J. Clin. Pathol..

[42] Ma Z., Wang L., Huang X., Ji H., Wang H., Yang Y., Ma Y., Chen J. (2023). Construction of the metabolism-related models for predicting prognosis and infiltrating immune phenotype in lung squamous cell carcinoma. J. Cancer.

[43] Lopez J.A., Noori T., Minson A., Li Jovanoska L., Thia K., Hildebrand M.S., Akhlaghi H., Darcy P.K., Kershaw M.H., Brown N.J. (2018). Bi-Allelic Mutations in STXBP2 Reveal a Complementary Role for STXBP1 in Cytotoxic Lymphocyte Killing. Front. Immunol..

[44] Subramanian A., Narayan R., Corsello S.M., Peck D.D., Natoli T.E., Lu X., Gould J., Davis J.F., Tubelli A.A., Asiedu J.K. (2017). A Next Generation Connectivity Map: L1000 Platform and the First 1,000,000 Profiles. Cell.

[45] Jabs D.A., Nussenblatt R.B., Rosenbaum J.T., Standardization of Uveitis Nomenclature SUN Working Group (2005). Standardization of uveitis nomenclature for reporting clinical data. Results of the First International Workshop. Am. J. Ophthalmol..

[46] International Study Group for Behçet's, D (1990). Criteria for diagnosis of Behcet's disease. Lancet.

[47] Sakata V.M., da Silva F.T., Hirata C.E., de Carvalho J.F., Yamamoto J.H. (2014). Diagnosis and classification of Vogt-Koyanagi-Harada disease. Autoimmun. Rev..

[48] Wu L., Zhou L., An J., Shao X., Zhang H., Wang C., Zhao G., Chen S., Cui X., Zhang X. (2023). Comprehensive profiling of extracellular vesicles in uveitis and scleritis enables biomarker discovery and mechanism exploration. J. Transl. Med..

